# Adrenocortical Carcinoma With Right Atrial Extension in a Three-Year-Old Child: A Case Report

**DOI:** 10.7759/cureus.101358

**Published:** 2026-01-12

**Authors:** Maymona AlShoaibi, Sara Hammedalnil, Mohamed Aljughaiman, Ghada S Abdullah

**Affiliations:** 1 Pediatric Cardiology, King Salman Heart Center, King Fahad Medical City, Riyadh, SAU; 2 Pediatrics, King Fahad Medical City, Riyadh, SAU; 3 Pediatric Cardiac Surgery, King Salman Heart Center, King Fahad Medical City, Riyadh, SAU; 4 Pediatric Cadiology, King Fahad Medical City, Riyadh, SAU

**Keywords:** adrenocortical carcinoma (acc), il-fraumeni syndrome, inferior vena cava (ivc) thrombosis, pediatric, right atrial tumor extension, tp53 mutation

## Abstract

Pediatric adrenocortical carcinoma is a rare and aggressive malignancy frequently associated with germline tumor protein p53 (TP53) mutations and can mimic congenital adrenal hyperplasia (CAH). We present a three-year-old child with progressive virilization, initially presumed to be CAH, who presented in adrenal crisis with abdominal distention. Imaging revealed a large right adrenal mass, with the mass extending from the right hepatic vein through the inferior vena cava (IVC) and into the right atrium, causing an almost complete obstruction of the tricuspid valve. After evaluation and stabilization, urgent surgery was performed, where the intracardiac mass was successfully removed, but the adrenal tumor remained due to anatomical complexity. Postoperative evaluation revealed a near-normal return of cardiac function, while the adrenal tumor continued to progress, and multiple emboli continued to form. Genetic testing revealed a TP53 mutation consistent with Li-Fraumeni syndrome. This case highlights the difficulties in differentiating between adrenocortical carcinoma and CAH in children. The rare presence of intracardiac extension of the tumor increases the challenge in managing these cases.

## Introduction

Pediatric adrenocortical tumors (ACTs) are rare malignancies, accounting for less than 0.2% of all childhood cancers, approximately 0.2-0.3 cases per million children per year. [1] These tumors are frequently associated with germline tumor protein p53 (TP53) mutations or cancer predisposition syndromes such as Li-Fraumeni and Beckwith-Wiedemann syndromes [1,2]. Early-stage disease can often be managed successfully with complete surgical resection. Advanced-stage ACTs are notoriously difficult to treat and carry a poor prognosis, with five-year survival rates ranging from 15-35% in metastatic cases [3]. Vascular invasion is a known hallmark of aggressive ACT, most commonly involving the renal vein and inferior vena cava (IVC) [1,4,5]. However, direct extension into the right atrium is exceedingly rare in the pediatric population and poses unique diagnostic and surgical challenges, sometimes requiring cardiopulmonary bypass [1]. Isolated reports have described successful resections of such extensive tumor thrombus, including a case in a nine-year-old boy [6]. We present a case of a three-year-old boy with adrenocortical carcinoma extending into the right atrium, highlighting a rare but critical scenario that requires multidisciplinary surgical planning and tailored oncologic management.

## Case presentation

A three-year-old male patient presented to the emergency department as a lifesaving referral in a hemodynamic crisis. He was diagnosed with late-onset congenital adrenal hyperplasia (CAH) five months prior at a different institution. He presented with the classical picture of adrenal crisis with hypotension, tachycardia, hypoglycemia, vomiting, and normal anion gap metabolic acidosis. Physical examination revealed a distended abdomen with a palpable left liver lobe. Genital examination revealed Tanner stage IV genitalia and coarse pubic hair with adult pattern distribution.

Upon reviewing the history, the presentation had started a year ago with rapidly progressive signs of precocious puberty, specifically axillary and pubic hair development. Following investigations, he was diagnosed with a case of late-onset CAH at a different institution, where he was started on hydrocortisone. 

The patient's presentation prompted multiple investigations, including an abdominal ultrasound, which showed a heterogenous right suprarenal mass measuring (7.1 x 10.4 x 7.1 cm) with internal calcification, and a thrombus extending from the right hepatic vein into the IVC and the right atrium. These findings raised doubts about the initial CAH diagnosis, and a full malignancy workup was requested.

**Figure 1:**
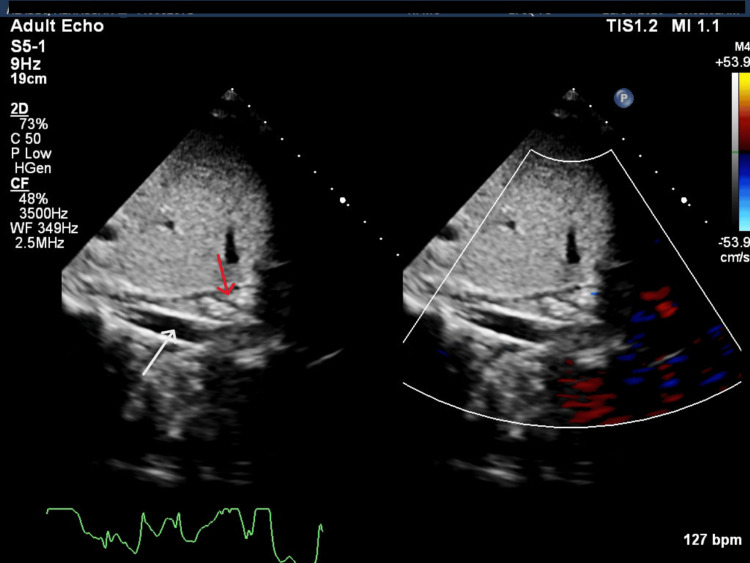
Subcostal echocardiographic view demonstrating the inferior vena cava (IVC) Subcostal echocardiographic view showing major abdominal vessels. The white arrow indicates the aorta, while the red arrow indicates the obliterated IVC.

An echocardiogram was done, which showed an echogenic shadow at the hepatic vein. The IVC visualization was limited due to mass effect (Figure 1).

**Figure 2:**
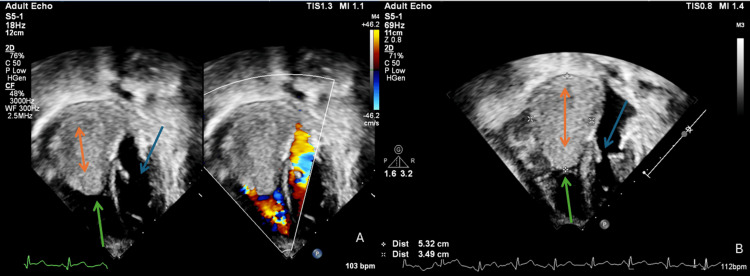
Preoperative four chamber echocardiogram of the heart Preoperative transthoracic echocardiography demonstrating intracardiac tumor extension. (A) Apical four chamber view showing large mass occupying the right atrium and obstructing the tricuspid valve. (B) Measurements of the mass (5.32 x 3.49 cm) as seen from the four chamber view of the echocardiogram. The blue arrow in both figures indicates a clear and unobstructed mitral valve, while the green arrows show an obstructed tricuspid valve, and the orange arrows show the tumor.

A huge right atrial mass was visualized, measuring 5.32 x 3.49 cm, extending into the right ventricular inflow leading to a near complete obstruction of the tricuspid valve and significantly compromising diastolic filling, as seen in Figures 2A, 2B.

**Figure 3:**
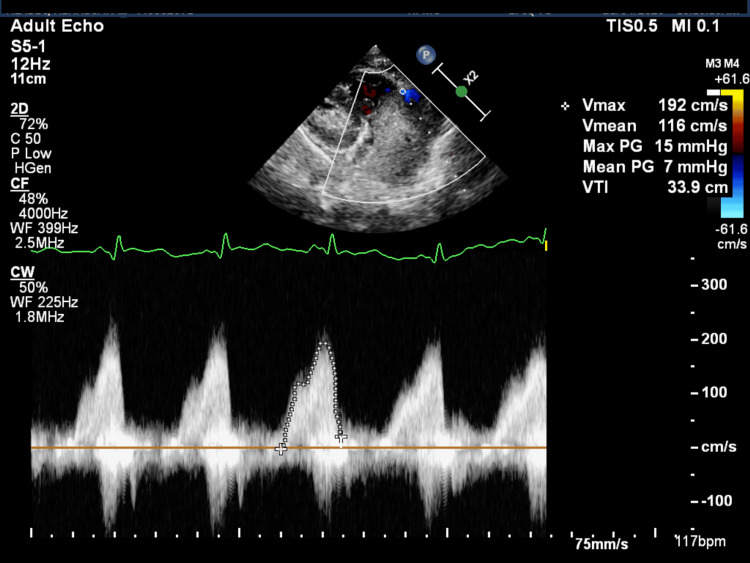
Parasternal long axis view of the heart with a continuous doppler The mean gradient across the tricuspid valve is seen in the echocardiogram above, showing a value of 7 mmHg, with a maximum of 15 mmHg, which is well above the normal expected gradient of <2 mmHg denoting severe obstruction.

The pressure gradient across the tricuspid valve was 7 mmHg (Figure 3).

The mass appeared to cross into the left atrium due to a possible inter-arterial septal defect. There was no left atrial dilation, and the remaining valvular function was normal. The right and left ventricular outflow tracts were patent, and left ventricular systolic function was normal (fractional shortening or FS=30.5%, and an ejection fraction or EF=60%). 

In light of the echocardiographic findings and the patient’s clinical presentation, the structure initially interpreted as a thrombus on ultrasound was suspected to represent an intracardiac extension of the adrenal mass rather than an isolated thrombosis.

Lab results showed low adrenocorticotropic hormone (ACTH) levels and high levels of 17-hydroxyprogesterone, testosterone, and dehydroepiandrosterone (DHEA). There were also signs of hepatic dysfunction with markedly elevated liver function tests, hyperbilirubinemia, and hypoalbuminemia, along with signs of coagulopathy. The patient also showed evidence of acute kidney injury with high creatine and urea, and oliguria. All the lab values can be seen in Tables 1-3.

**Table 1:** Endocrine investigations at presentation ACTH: Adrenocorticotropic Hormone; DHEA: dehydroepiandrosterone

Parameter	Value	Units	Reference range
ACTH	1.05	pmol/L	1.6-13.9
17-hydroxyprogesterone	10.77	ng/mL	in children 3-6 years: <2.77
Testosterone	30.17	nmol/L	Prepubertal <0.5
DHEA	77.94	µg/dL	Prepubertal <40

**Table 2:** Hepatic panel and coagulation parameters at presentation AST: Aspartate Aminotransferase; ALT: Alanine Aminotransferase; GGT: Gamma-Glutamyl Transferase; PT: Prothrombin Time; INR: International Normalized Ratio

Parameter	Value	Units	Reference Range
AST	>4001	U/L	18-36
ALT	6118	U/L	9-25
GGT	37	U/L	6-16
Total bilirubin	13.9	µmol/L	6.1-15.3
Direct bilirubin	7	µmol/L	≤3.4
Albumin	28.1	g/L	38-47
PT	53.4	sec	11.9-15.9
INR	4.52	—	0.87-1.16
Ammonia	260	µmol/L	18-72

**Table 3:** Chemistry and electrolyte panel at presentation

Parameter	Value	Units	Reference range
Creatinine	109	µmol/L	18-38
Urea	6.5	mmol/L	2.5-6.0
Potassium	6.7	mmol/L	3.4-4.7
Sodium	140	mmol/L	136-145
CO₂ (HCO₃⁻)	12	mmol/L	14-24
Anion gap	24.7	mmol/L	~12 ± 4
Chloride	110	mmol/L	98-107
Calcium	2.09	mmol/L	2.20-2.50
Phosphorus	1.98	mmol/L	1.33-1.92

A multidisciplinary team meeting was held, involving primary care teams and surgical specialties to determine the next steps. Given his hemodynamic instability, echocardiography results, and signs of multiorgan dysfunction, concern was raised about his ability to withstand the anesthesia induction and contrast dye for CT/MRI. After careful deliberation, the team decided to defer imaging as the patient was deemed high risk for cardiovascular collapse with anesthesia induction.

The next point of discussion was about the imaging findings. Based on the findings of the ultrasound and echocardiography, along with the presentation, the initial diagnosis of late-onset CAH was unlikely. The mass was instead presumed to be an androgen secreting tumor with radiological features aligning more with adrenocortical carcinoma but confirming this was difficult without a biopsy sample or further imaging. There were also concerning features of compression of hepatic structures and further cephalad extension.

The discussion was shifted towards possible surgical intervention, which posed its own risks given the size and location of both masses, and proximity to vital structures. There was a high risk of massive intraoperative bleeding, injury to hepatic and renal vasculature, and a need for possible IVC patch reconstruction. Despite these risks, the team concluded that surgical debulking of both the cardiac and suprarenal mass, performed in a single combined operation, would offer the patient the best chance of survival.

On the fourth day of admission, the patient went in for surgery and was placed on bypass. On visualization of the right atrium, the tumor looked large and fragile. It was invading the junction between the IVC and the right atrium, along with the walls of the IVC and hepatic veins. The right atrial mass was removed in multiple pieces, and the entire cardiac portion was removed. Intermittent circulatory arrest was used to resect as much as possible of the mass in the hepatic vein and abdominal IVC. Intraoperative visualization revealed an extensive, fragmented adrenal tumor that was difficult to delineate, and no further surgical intervention was pursued.

The patient’s clinical course following the operation was long and complicated. He was critically ill and intubated with ionotropic support. By day two post-op, there were progressive signs of acute kidney injury, with anuria and elevated urea and creatinine. He was also exhibiting worsening generalized edema, along with chest congestion and pulmonary edema. Due to these findings, the patient started continuous renal replacement therapy (CRRT). 

**Figure 4:**
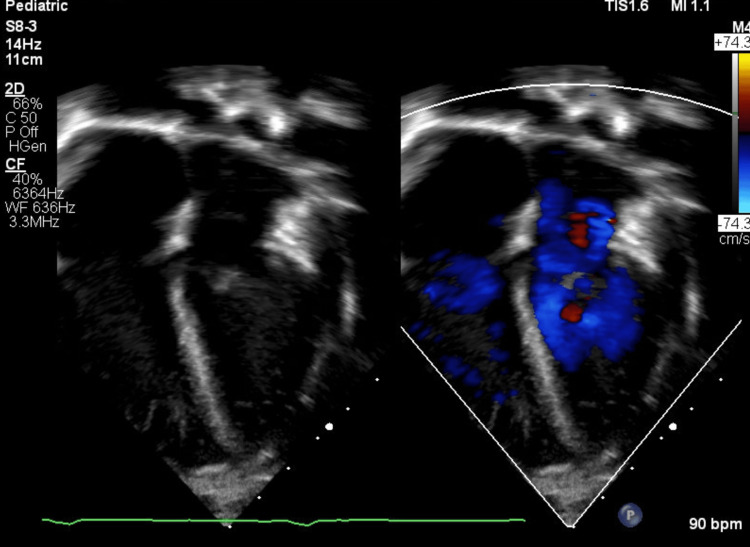
Post-operative four chamber view echocardiogram Post-operative echocardiogram showing a normal right atrium with no mass, and a normal tricuspid valve.

Despite the deteriorating clinical picture, the post operative echocardiography was reassuring. The mass was not visualized intracardially (Figure 4) and cardiac function was intact.

**Figure 5:**
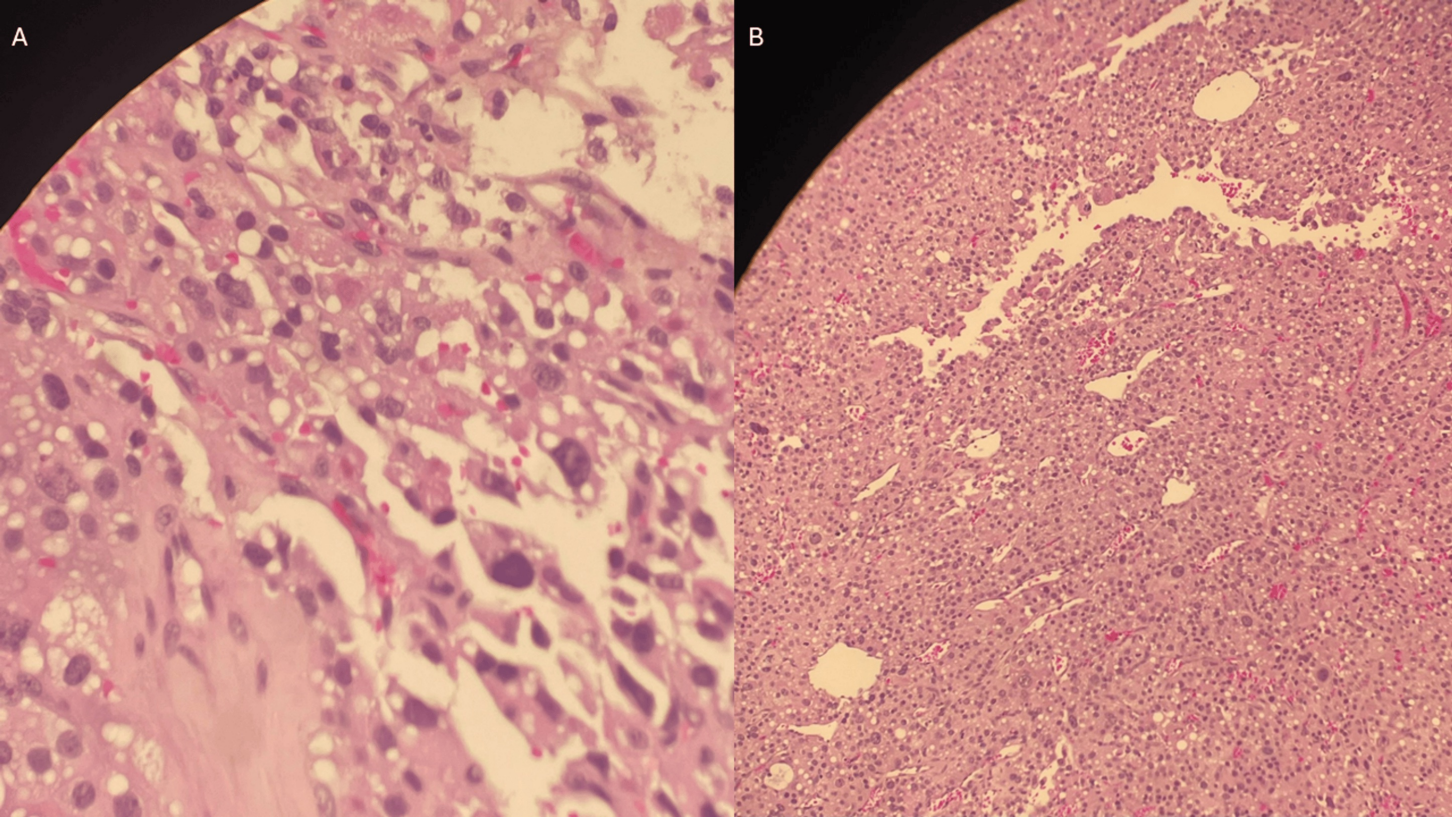
Histopathological sections of the right atrial mass biopsy 5A: This slide is a microscopic view showing pleomorphic cells with prominent cellular atypia; 5B: Slide seen is a microscopic view showing broad sheets of tumor with areas of necrosis. Both images demonstrate features consistent with adrenocortical carcinoma. Immunohistochemistry (not shown) demonstrated positivity for synaptophysin, Cluster of Differentiation (CD)99, Melanoma antigen recognized by T cells 1 (MART-1 or Melan-A), inhibin, CD56, vimentin, cytokeratin (CAM5.2; focal), and diffuse mutant-type p53, with negative staining for chromogranin, cytokeratin, and S-100.

Biopsy results of the resected cardiac tumor confirmed the diagnosis of adrenocortical carcinoma as seen in Figures 5A, 5B.

**Figure 6:**
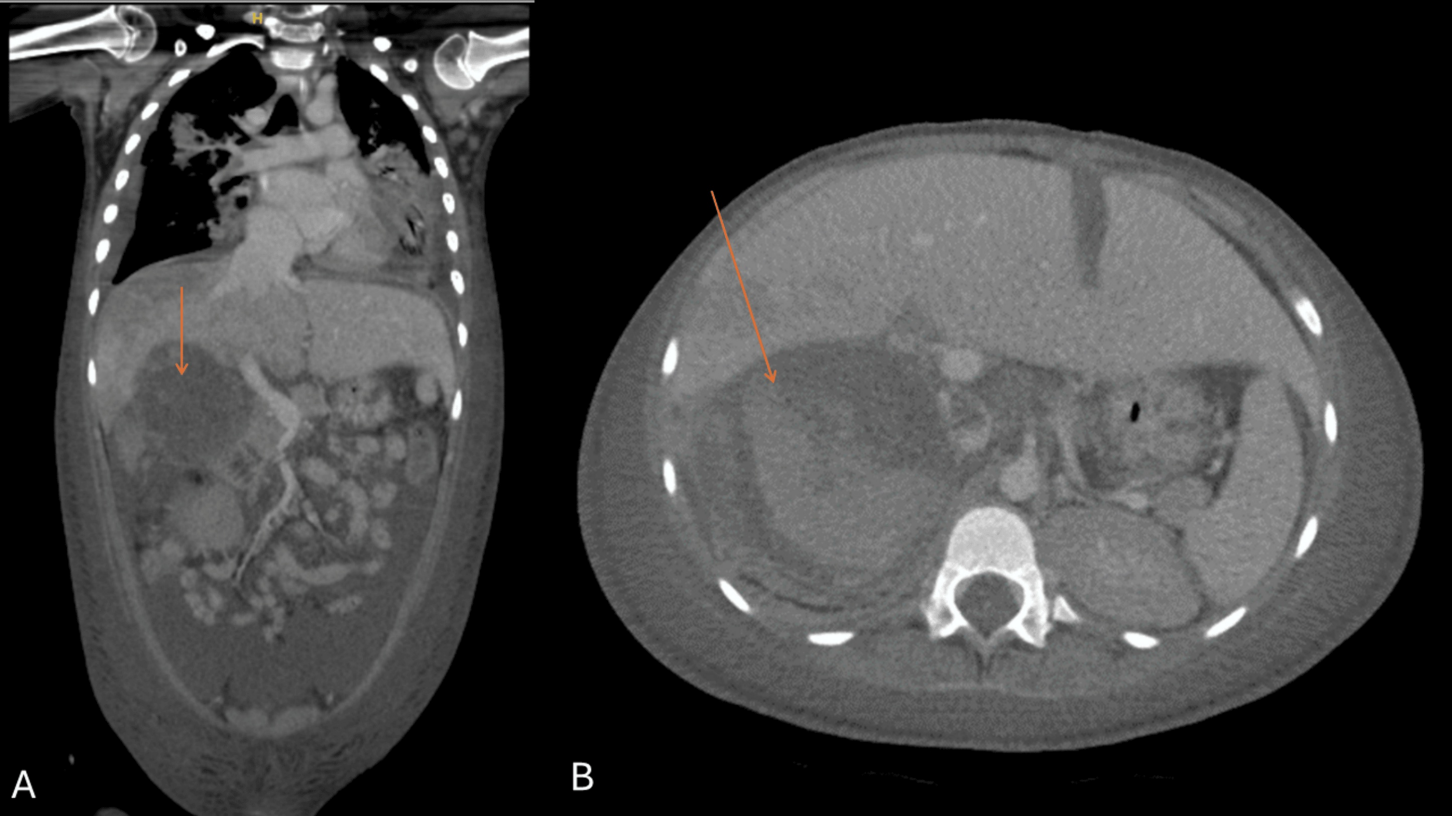
Contrast-enhanced full body CT scan showing the right adrenal mass A: coronal and B: axial view of the CT scan. Both show a right sided adrenal tumor, indicated by the orange arrow, demonstrating heterogenous enhancing and non-enhancing components.

Removal of the cardiac tumor slightly decreased the risks with anesthesia induction and a full body CT scan was done to stage the tumor (Figures 6A, 6B).

The adrenal tumor now measured 7.8 x 7.3 x 10.2 cm, with a hyperintensity suggestive of active bleeding, along with fat stranding indicating tumor rupture. Free fluid was noted in the abdomen and pelvis indicating hemoperitoneum.

Multiple emboli were identified. A wedge-shaped hepatic enhancement with no right hepatic vein opacification indicated hypoperfusion. There was also a large filling defect (1.5 x 1.1 x 2.2 cm) involving the infrahepatic segment of the IVC noted, suggestive of a thrombus. Bilateral renal hypoperfusion was noted, which could be due to the ongoing acute kidney injury or thrombus formation. Bilateral pleural effusion with patchy lung consolidation was also present, indicative of infarction or possible aspiration due to recent intubation.

Following the results of the CT, the tumor was deemed inoperable, and as the tumor was not sensitive to chemotherapy, management was shifted to palliative care. Given these decisions along with the patient’s deteriorating clinical status a Do-not-resuscitate (DNR) status was agreed upon and discussed with the family.

In the weeks following, the patient continued to deteriorate. He remained intubated and hemodynamically unstable. Acute kidney injury remained progressive despite CRRT and diuretic support. Due to prolonged steroid exposure, steroid taper was begun.

After multiple failed attempts, the patient was extubated two weeks post-operatively. He experienced multiple challenges surrounding cytokeratin and oxygenation. He was still anuric with creatinine levels still rising. Then 17 days after the operation, the patient’s heart rate fluctuated with low blood pressure, he continued to progressively deteriorate with persistent hypotension and hypoxia and eventually went into cardiopulmonary arrest. A whole exome study, sent pre-mortem, revealed a mutation in the TP53 gene weeks later, with a genetic diagnosis of autosomal dominant Li-Fraumeni syndrome providing an explanation for the development of an aggressive tumor in this patient.

## Discussion

Adrenocortical carcinoma in children is a rare and aggressive malignancy, comprising less than 0.2% of pediatric cancers [1]. Pediatric ACTs frequently present with signs of hormone hypersecretion, particularly virilization due to excess androgens, and are often linked to germline TP53 mutations, as seen in Li-Fraumeni syndrome [1,2]. In this case, the child presented with classic features of virilization, initially misdiagnosed as CAH, which delayed recognition of the underlying malignancy. This diagnostic pitfall is well-documented, as ACTs can mimic CAH biochemically due to similar steroidogenic profiles [3]. Despite this hormonal overlap, several clinical 'red flags' should prompt evaluation for malignancy, including rapidly progressive virilization, onset beyond infancy, poor response to glucocorticoid therapy, asymmetric or enlarging adrenal masses, and systemic features such as abdominal distention or weight loss, even in the presence of elevated 17-hydroxyprogesterone levels. 

What makes this case particularly unique is the tumor’s extensive vascular invasion into the IVC and the right atrium, with near-obstruction of the tricuspid valve, an exceptionally rare occurrence in pediatric patients. While IVC invasion is not uncommon in advanced ACT, right atrial extension has only been reported in a few isolated pediatric cases [4,5]. In such instances, cardiopulmonary bypass is often required for safe tumor debulking [1].

The management of this patient was extraordinarily complex due to multi-organ dysfunction, including hepatic injury, renal failure, coagulopathy, and adrenal insufficiency. The child underwent high-risk cardiac surgery, with removal of the intracardiac tumor mass under circulatory arrest. While the cardiac resection was successful, the adrenal mass remained unresectable due to anatomical complexity and intraoperative risks. Postoperative imaging confirmed progressive disease, including hemoperitoneum and thrombus recurrence, consistent with the aggressive nature of ACT, especially in the context of confirmed TP53 mutation.

Pediatric ACTs are notoriously chemoresistant. Although regimens including mitotane and platinum-based agents are used, their efficacy is limited, particularly in metastatic disease [1,6]. For this patient, surgical intervention was the only potentially curative option, and once complete resection was no longer feasible, the prognosis became poor. The five-year survival rate for stage IV pediatric ACT is dismal, around 15-35%, and even lower when resection is incomplete or delayed [3,6].

This case underscores the importance of maintaining high clinical suspicion for ACT in children presenting with virilization, especially when biochemical markers are discordant or the patient’s condition deteriorates despite standard CAH therapy. It also highlights the need for early imaging and multidisciplinary planning, particularly when vascular extension is suspected. Finally, this case adds to the limited body of literature describing cardiac extension in pediatric ACT, reinforcing its role as a potential surgical emergency with significant prognostic implications.

## Conclusions

This case highlights a rare and aggressive presentation of pediatric adrenocortical carcinoma with extensive vascular invasion reaching the right atrium. It underscores the diagnostic challenges of distinguishing ACT from CAH, especially when initial hormonal findings overlap. The presence of cardiac extension in such a young child is exceptionally uncommon and carries significant surgical and prognostic implications. This case emphasizes the importance of early recognition, multidisciplinary management, and genetic testing for TP53 mutations, which may guide surveillance strategies in affected families and facilitate earlier diagnosis, and are crucial in managing such complex cases. 
